# Does WASH FIT Improve Water, Sanitation, and Hygiene and Related Health Impacts in Healthcare Facilities? A Systematic Review

**DOI:** 10.3390/ijerph22050708

**Published:** 2025-04-30

**Authors:** Hannah Lineberger, Ryan Cronk, Sena Kpodzro, Aaron Salzberg, Darcy M. Anderson

**Affiliations:** The Water Institute, Gillings School of Global Public Health, The University of North Carolina at Chapel Hill, 4114 McGavran-Greenberg Hall, CB #7431, Chapel Hill, NC 27516, USA

**Keywords:** water and sanitation for health facility improvement tool, hygiene, waste management, environmental health services, low- and middle-income countries

## Abstract

Environmental health services in healthcare facilities are important, but coverage gaps remain. The World Health Organization and UNICEF developed the Water and Sanitation for Health Facility Improvement Tool (WASH FIT) to assess and improve environmental health services in healthcare facilities. Over 70 countries have adopted it. However, there is little evidence of its effectiveness. This systematic review evaluates whether WASH FIT improves environmental health services and associated health outcomes and impacts. We extracted data from 31 studies on inputs, activities, outputs, outcomes, and impacts associated with WASH FIT and summarized findings using a logic model framework. Twenty-three studies reported quantitative outputs for environmental health services. Of these, only nine reported longitudinal changes in these outputs throughout WASH FIT implementation. Six studies reported quantitative outcomes; the remainder described outcomes qualitatively or not at all. No studies directly measured impacts or evaluated WASH FIT against a rigorous control group. We found that available evidence was insufficient to evaluate WASH FIT’s effectiveness. Further effort is needed to identify the inputs and activities required to implement WASH FIT and to draw specific links between changes in outputs, outcomes, and impacts. Opportunities exist to improve evidence by more comprehensively reporting WASH FIT assessments and exploiting data on health impacts within health management information systems.

## 1. Introduction

Environmental health services, including water, sanitation, hygiene, cleaning, and waste management, are critical for safe healthcare facilities. They reduce the risk of healthcare-associated infections, which in turn reduces antimicrobial resistance and healthcare costs [1,2]. Environmental health services are associated with improved quality of care, care seeking, and patient and healthcare worker satisfaction [3,4,5,6]. Investing in environmental health services in healthcare facilities is recommended as an important intervention to improve maternal and child health and health system resilience [7].

Despite the benefits of environmental health services, coverage gaps remain. Overall, 22% of healthcare facilities globally lack basic water services, 49% lack basic hygiene services, and 10% lack basic sanitation services [8]. Healthcare facilities in low- and middle-income countries experience the largest deficiencies and disproportionate infectious disease burdens, contributing to a cycle of poverty and inequitable health outcomes [8,9].

To progress towards universal access, the World Health Organization (WHO) and the United Nations Children’s Fund (UNICEF) developed the Water and Sanitation for Health Facility Improvement Tool (WASH FIT) in 2017 [10], with a second edition released in 2022 [11]. WASH FIT is a continuous quality improvement tool to help evaluate and improve environmental health services in healthcare facilities and is predominantly used in low- and middle-income countries.

WASH FIT implementation follows a five-step cycle: (1) establish and train a WASH FIT team and document decisions; (2) assess the facility; (3) identify and prioritize areas for improvement; (4) develop an improvement plan and act; and (5) monitor, review, adapt, and improve. This cycle is intended to be iterated every 6–12 months for continuous quality improvement. The cycle has no definitive endline and is intended to be incorporated as a sustained component of ongoing facility operation. The WASH FIT manual suggests that team members may include facility managers, medical staff (e.g., nurses, doctors), engineers or technicians, and community or district authorities (e.g., district health officers, community leaders, patient groups), depending on the specific healthcare facility type and locally relevant stakeholders. Extensive training materials are available through the WHO/UNICEF webpage [12] (see, e.g., the WASH FIT training manual [13]). Training materials predominantly provide guidance on how to conduct prescribed activities for each step of the WASH FIT cycle.

Integrated into WASH FIT is an assessment tool containing indicators to assess coverage and access to environmental health services. This assessment tool is used in Step 2 of the WASH FIT cycle. These indicators guide improvement plans (Step 3) and are intended to be monitored and reassessed (Step 5) to evaluate progress as part of continuous quality improvement. The WASH FIT first edition assessment tool comprises 66 indicators across four core domains: water, sanitation (divided into Part A for “sanitation” and Part B for “healthcare waste”), hygiene (divided into Part A for “hand hygiene” and Part B for facility environment, cleanliness, and disinfection), and management [10]. The second edition comprises 95 indicators across five primary domains (water, sanitation, healthcare waste management, hand hygiene, environmental cleaning) and two secondary domains needed to support environmental health services infrastructure and practices (energy and the environment; management and workforce); two cross-cutting themes (climate resilience and equity and inclusiveness) are integrated across these seven domains [11]. Indicators assess the availability of infrastructure (e.g., presence of water and energy sources), behavioral practices (e.g., waste segregation), and managerial factors (e.g., training and audits for waste management and hygiene practices, presence of standard operating procedures). Specific indicator lists can be found in the appendices of the respective editions of the WASH FIT manuals [10,11].

WASH FIT has been implemented in over 70 countries as of 2025 and globally is one of the most widely adopted and influential tools for prioritizing investments in environmental health services in healthcare facilities [14]. At least 13 countries have adopted WASH FIT as a core component of national strategies for environmental health services in healthcare facilities [14], and individual country programs have received investments of millions of dollars from bilateral and foundation donors to implement WASH FIT programs (e.g., in Mali [15] and Ghana [16]).

Yet little is known about WASH FIT’s effectiveness in changing environmental health service outputs and associated health outcomes and impacts—nor has a robust theory of change been proposed through which WASH FIT may achieve impact. Most research on WASH FIT describes its use as a one-time assessment tool to identify service gaps and prioritize resources [17,18,19]. The WASH FIT manual itself proposes theories of change that hypothesizes WASH FIT impact pathways [11], as does a study by authors affiliated with the WHO [20], but neither has been empirically vetted.

We conducted a systematic review to evaluate whether WASH FIT improves outputs for environmental health services and associated health outcomes and impacts. Our objectives were to (1) describe the study designs and WASH FIT assessment domains measured in WASH FIT studies; (2) document the inputs and activities required to deliver WASH FIT; and (3) evaluate the effect of WASH FIT on outputs, outcomes, and impacts.

## 2. Material and Methods

### 2.1. Study Overview

We systematically reviewed peer-reviewed and gray literature on WASH FIT implementation and evaluation. We extracted data on WASH FIT study designs and locations, length of WASH FIT implementation, and WASH FIT assessment domains measured in each study. We extracted data on WASH FIT inputs and activities; we then mapped activities to the WASH FIT’s intended five-step cycle [11] and inductively developed categories for inputs based on observed similarities in the data. We used a similar approach to compile and evaluate WASH FIT outputs, outcomes, and impacts.

We have complied with PRISMA reporting guidelines for this manuscript [21]; the checklist is provided in Supplemental File S1.

### 2.2. Conceptual Framework

**Figure 1:**
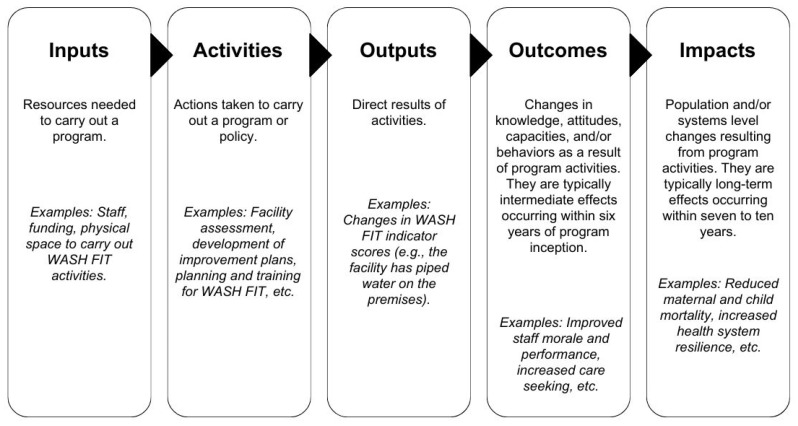
Defining components of the logic model. Adapted from the Kellogg Foundation [22].

We used a logic model to guide our data extraction, analysis, and narrative synthesis (Figure 1). Logic models visually depict the relationships between an intervention’s inputs, activities, outputs, outcomes, and impacts [22]. Logic models are an important tool for program planning and evaluation. They identify required inputs and activities to understand if programs are being delivered as intended. They identify expected outputs, outcomes, and impacts, which can support impact evaluations to understand if programs are working as intended. Furthermore, logic models can help understand a program’s theory of change (i.e., theory of how and why a program creates change) by identifying specific pathways by which inputs and activities yield short-term outputs, intermediate outcomes, and long-term impacts [22,23,24].

We used a logic model to document the inputs and activities required to deliver WASH FIT [22]. We assessed changes in outputs, outcomes, and impacts within the logic model framework to evaluate WASH FIT effectiveness.

### 2.3. Search Strategy

We conducted systematic searches of peer-reviewed and gray literature. We performed database searches in PubMed and Scopus for peer-reviewed literature on 13 February 2024. We searched by title and abstract in PubMed and title, abstract, and keyword in Scopus for four terms: “WASH FIT”, “WASH FAST”, “Water and sanitation for health facility improvement tool”, and “Water and sanitation for facility improvement tool”. We restricted our search to studies published in 2017 or later (the publication year for the first edition of WASH FIT).

For gray literature, we searched washinhcf.org, the largest online repository of resources related to environmental health services in healthcare facilities, curated by the WHO and UNICEF. Resources in the repository are tagged with keywords describing their content when archived. We searched washinhcf.org on 6 February 2024 for all resources tagged as WASH FIT. We identified additional references through a 2022 report on WASH FIT case studies [25] and consultation with experts from the WHO and UNICEF.

### 2.4. Inclusion and Exclusion Criteria

We included studies that reported WASH FIT implementation in a healthcare facility in a low- or middle-income country and described at least one component of the logic model (i.e., inputs, activities, outputs, outcomes, or impacts) related to WASH FIT. Studies of all designs that used either the first or second edition of WASH FIT were eligible for inclusion. We included only studies written in English, French, or Spanish. To be eligible for inclusion, we required that study report implementing at least one step of the WASH FIT cycle in a healthcare facility. For example, we included some studies that partially implemented the WASH FIT cycle but did not report all five steps. We excluded studies that reported solely on national-level actions (e.g., national-level training of trainers) but never described WASH FIT implementation in a healthcare facility. A single author screened all articles.

### 2.5. Data Extraction and Synthesis

We first developed, piloted, and revised the extraction form. We developed a preliminary form in Microsoft Excel, guided by the logic model framework. Two authors piloted the form on five studies that were included in the search, met to discuss the challenges and shortcomings of the extraction form, and revised it accordingly to create the final extraction form. The final extraction form included the following categories: study design and context (e.g., location, healthcare facility characteristics), WASH FIT assessment domains measured, WASH FIT inputs and activities, and WASH FIT outputs, outcomes, and impacts. The final extraction form is provided in Supplemental Information File S2. Once the extraction form was finalized, all studies included in the search were extracted by a single author.

We used narrative synthesis to address our three objectives. To describe the study designs and WASH FIT assessment domains, we described similarities and differences in which WASH FIT domains each study measured and reported. To document inputs and activities, we organized extracted data in the logic model framework and inductively developed categories for inputs based on observed similarities in the extracted data. We followed the same approach to document outputs, outcomes, and impacts. There were insufficient data for a meta-analysis, so we summarized our findings using narrative synthesis.

## 3. Results

### 3.1. Included Studies

**Figure 2:**
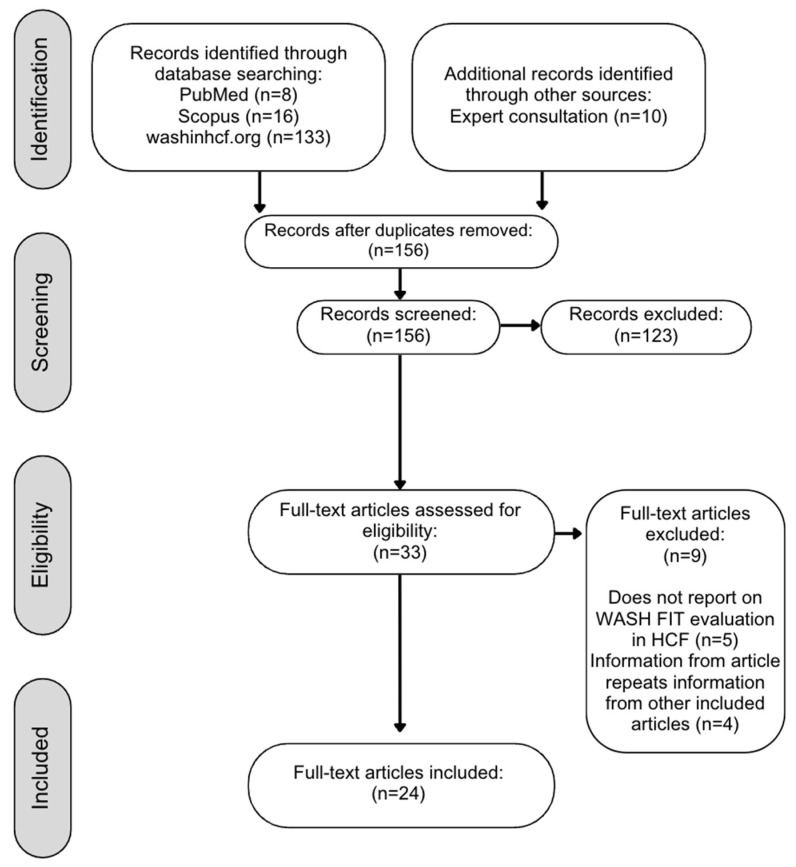
Flow chart of studies included in a review of WASH FIT effectiveness.

Our search yielded 156 unique articles (Figure 2). We identified 17 studies that were not identical but reported data on the same WASH FIT program (typically a gray literature report subsequently published in peer-reviewed literature). We included multiple studies on the same WASH FIT program only when they reported different data for inputs, activities, outputs, outcomes, and/or impacts. Where articles presented the same data, we excluded the older or less comprehensive version. Details of unique articles excluded based on duplicate data are provided in Supplemental Information File S2.

In total, 24 articles met our inclusion criteria [15,17,18,19,25,26,27,28,29,30,31,32,33,34,35,36,37,38,39,40,41,42,43,44]. One article was a WHO report containing eight eligible case studies, each summarizing WASH FIT implementation in a different low- or middle-income country [25]. We extracted and reported these case studies separately in our results for 31 studies included in our synthesis.

**Figure 3:**
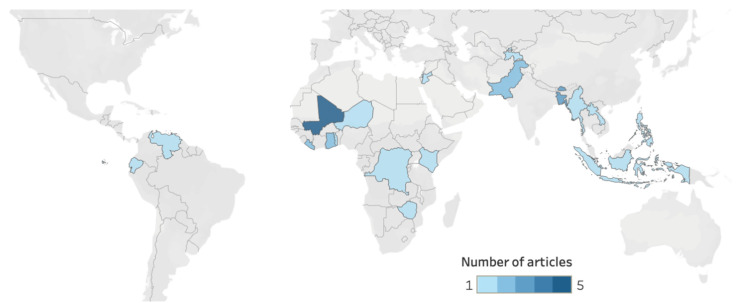
Geographic distribution of WASH FIT studies. Cropped regions of the map did not have any countries with WASH FIT studies. Countries in grey had no studies.

We found published reports on WASH FIT implementation and evaluation in 20 countries (Figure 3). Studies from the WHO African Region (n = 14, 45%) and South-East Asian Region (n = 9, 29%) represented 74% (n = 23) of the studies.

More than half of the included studies (n = 17, 55%) reported implementing WASH FIT in ten or more healthcare facilities, but sample sizes varied from 1 to 256. Of studies that reported healthcare facilities’ characteristics, WASH FIT implementation spanned all healthcare facility types (i.e., primary, secondary, tertiary), both public and private ownership, and diverse types of health service provision, including inpatient, outpatient, maternity, neonatal, pediatric, medical, surgical, family planning, and laboratory services. The reporting of healthcare facility size varied. Some studies reported the number of beds (n = 4, 13%), some reported the number of patient consultations (n = 3, 10%), and others reported both (n = 2, 6%). Fourteen studies (45%) reported no healthcare facility characteristics besides facility type or location. Of studies that reported location (n = 14, 45%), 11 included HCFs in rural areas.

We identified ten studies of WASH FIT implementation in special settings or situations: three in refugee camps [25,30,34] and seven as part of pandemic response or recovery for COVID-19 [17,19,25,39], Ebola [31], or cholera [35].

### 3.2. Study Designs and WASH FIT Implementation Duration

Study designs comprised case study (n = 17), cross-sectional (n = 8), and quasi-experimental (n = 6). Not all studies reported the length of WASH FIT implementation. Of those that did report implementation duration (n = 19, 61%), the duration ranged from two days to three years, with most (n = 11) reporting implementation for one year or less. Studies with implementation lasting only days or months typically used WASH FIT as a one-time assessment tool to identify gaps in environmental health service provision, set priorities, and inform the development of improvement plans. Nine studies (29%) recorded at least two rounds of WASH FIT assessment and systematically compared scores over time. Twelve studies (39%) completed one WASH FIT assessment to assess pre-improvement scores, with no follow up assessments. The remaining ten studies (32%) did not specify how many assessments were conducted. Details of specific durations of implementation and number of assessment rounds for each study can be found in Supplemental Information File S2.

### 3.3. Evaluation of WASH FIT Domains and Indicators

**Figure 4:**
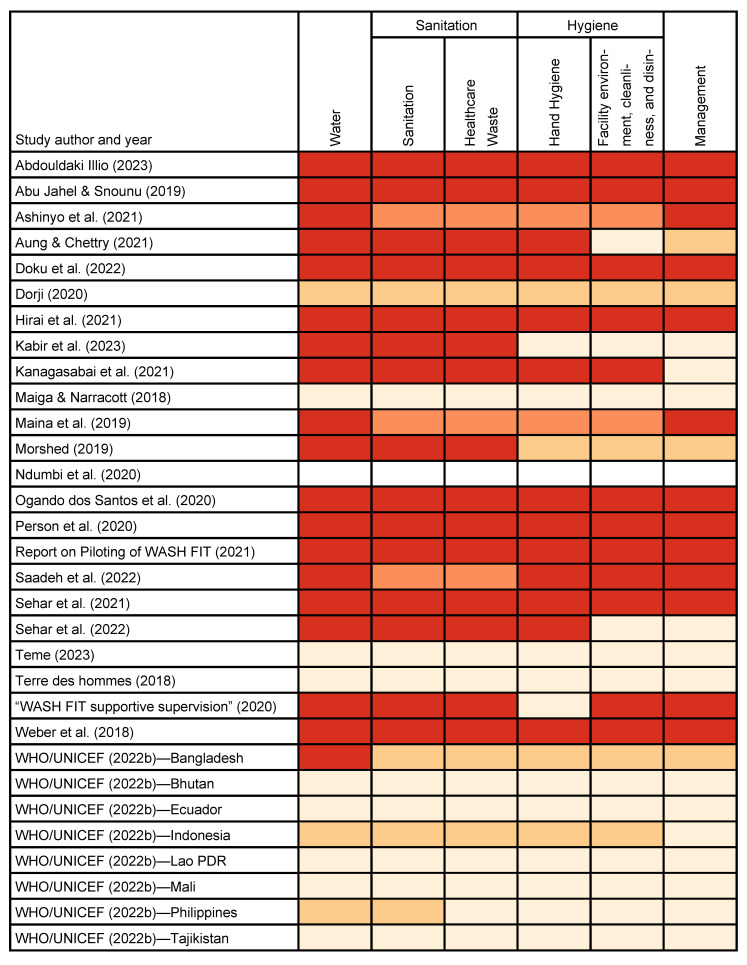

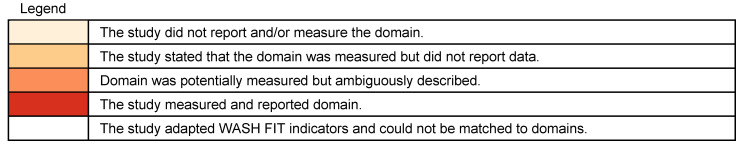
WASH FIT domains assessed in WASH FIT Step 2 (assess the facility) for studies included in the review.

In all studies, WASH FIT implementation and data collection were carried out before 2022 (the release date for the second edition), so we determined that all studies used the first edition. The aggregate “hygiene” and “sanitation” domains for the assessment tool in the first edition of WASH FIT complicated our efforts to extract information on the specific WASH FIT domains reported by each study. Fourteen studies reported disaggregated indicators for “hand hygiene” versus “facility environment, cleaning, and disinfection”. In contrast, two studies generically reported that they measured “hygiene” but did not specifically describe whether they measured “hand hygiene” indicators, “facility environment” indicators, or both. Similarly, 15 studies reported disaggregated indicators for “sanitation”. Three studies generically reported that they measured “sanitation” but did not specify whether they measured only “sanitation” or both “sanitation” and “healthcare waste” (Figure 4).

Nine articles reported on all WASH FIT first edition domains and sub-domains. For studies that did not, the most commonly excluded were management and hand hygiene. Not all studies explicitly reported the total number of WASH FIT indicators measured. Among those that did (n = 16, 52%), the number of indicators ranged from seven to 67. Eight studies (26%) adapted WASH FIT indicators or supplemented them with indicators from other sources. For example, Ndumbi et al. [35] reported using WASH FIT, but the indicators did not directly match any of the first edition WASH FIT indicators. Saadeh et al. [39] measured 150 indicators, including WASH FIT and other indicators for infection prevention and control, COVID-19 safety, and pharmacy safety.

### 3.4. Inputs and Activities Required to Deliver WASH FIT

**Figure 5:**
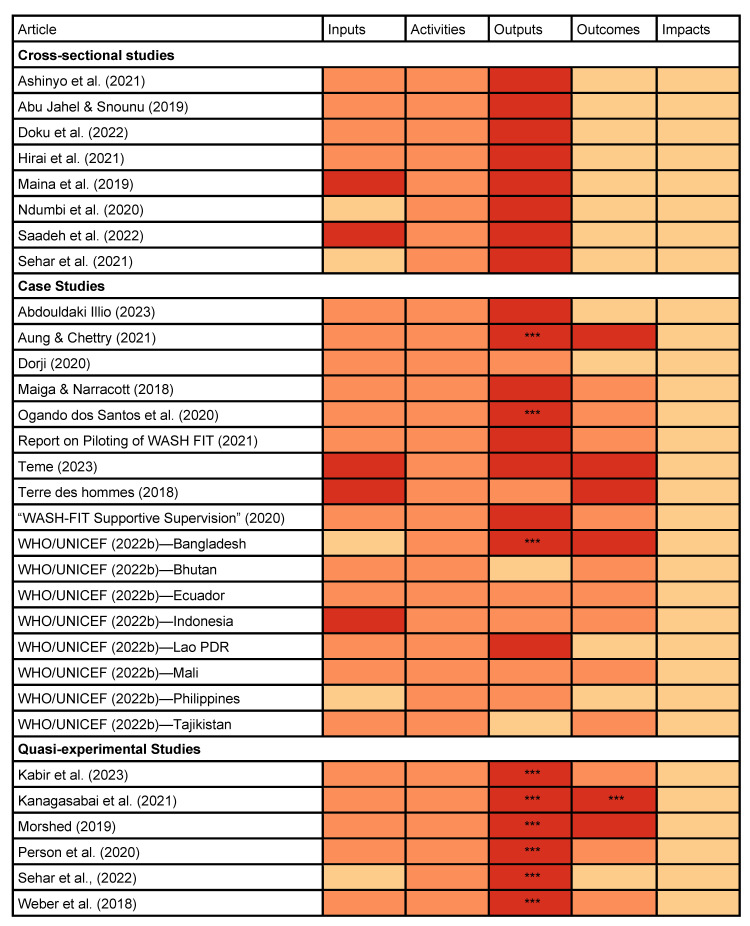

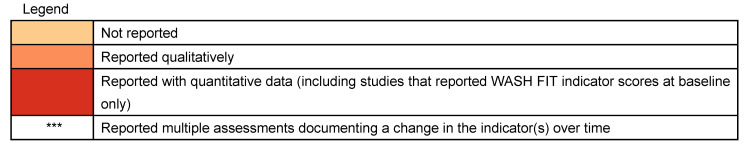
Reporting of inputs, activities, outputs, outcomes, and impacts among WASH FIT studies.

Approximately 84% (n = 26) of studies reported inputs and 100% (n = 31) reported activities. Among these, most reported inputs and activities qualitatively. Case studies more commonly reported inputs than cross-sectional studies (Figure 5).

After reviewing similarities in our extracted data, we inductively developed the following input categories: financial support, government support, human resources, and technical assistance (Table 1). Financial support and human resources were reported both qualitatively and quantitatively. Five studies reporting specific funding amounts or numbers of staff [15,33,39,42,45]. Others reported inputs qualitatively, though the level of detail varied. One commonly described input was “support” or “technical assistance” from government ministries or development partners, though often the specific nature of the support or technical assistance was not described.

Most studies reported activities qualitatively as well, describing the actions taken to implement WASH FIT. Studies did not directly tie activities to the WASH FIT cycle. Still, most reported activities could be mapped to the five steps in the cycle (i.e., establishing and training the WASH FIT team, assessing healthcare facilities, identifying and prioritizing areas for improvement, developing an improvement plan, and acting, monitoring, reviewing, adapting, and improving). 

We also identified a commonly reported activity that was not included in the WASH FIT cycle but was suggested as an optional preparation in the WASH FIT guide: adaptation. Ten studies reported adapting WASH FIT to local contexts or current needs. For example, the WASH FIT team in the Philippines consulted with stakeholders to harmonize WASH FIT indicators with national policies and guidelines [25]. One WASH FIT program in Venezuela translated and adapted WASH FIT material prior to implementation [36], and another in Mali integrated 12 indicators from a COVID-19 scorecard into WASH FIT [42].

### 3.5. Outputs, Outcomes, and Impacts of WASH FIT

Most studies reported quantitative outputs related to WASH FIT implementation (n = 23, 74%) (Figure 5). Outcomes were reported less frequently. Overall, 83% (n = 5) of quasi-experimental studies, 76% (n = 13) of case studies, and no cross-sectional studies reported outcomes. One case study reported qualitatively that staff perceived a reduction in “communicable diseases” after WASH FIT [34], but no study directly measured impacts. Based on observed similarities in our extracted data, we identified four output categories and seven outcome categories (Table 2).

We classified WASH FIT assessment indicators as output indicators because these indicators are designed to be assessed in Step 2 to inform improvement plans and then re-assessed in Step 5 to evaluate performance of the improvement plan. Most studies in our review reported conducting a facility assessment as Step 2 of the WASH FIT cycle. Six quasi-experimental studies [30,31,34,37,40,44] and three case studies [28,36,43] assessed and reported changes in WASH FIT indicator scores over time. We identified an additional output indicator not included in the WASH FIT assessment tool: personnel trained on WASH FIT. Studies presented data about personnel trained on WASH FIT in varied ways, with some reporting the number of personnel trained while others only shared that an unspecified number received training.

Outcomes varied more widely than outputs and were less commonly reported quantitatively. Ten studies reported that WASH FIT contributed to policy changes or informed governments’ decisions to scale up WASH FIT. Four studies reported that WASH FIT implementation helped improve collaboration with government entities. Seven studies reported that WASH FIT helped justify allocated budgets for environmental health services. Twelve studies also noted improvements in occupational safety or knowledge, attitudes, or practices among healthcare staff, patients, or community members. Most of these changes were reported qualitatively through interviews or observations. Finally, four studies indicated that WASH FIT helped improve community engagement by creating new avenues and incentives for healthcare workers to engage with community members.

No studies rigorously reported impacts. One case study reported interview debriefing with program staff, stating that “two-thirds of the interviewees cited that there is a lower incidence of communicable diseases among the staff” [34]. However, the methods did not specify how, if at all, communicable disease incidence was directly measured. Most studies referred to a connection between improved WASH outputs and various long-term impacts to justify the importance of WASH FIT, including reduced healthcare-associated infections [39,44], antimicrobial resistance [19,41], infectious disease transmission [17], and healthcare costs [17,34] as well as improved quality of care [19,40], health-seeking behaviors [17,19], and maternal and child health [31]. However, these hypothesized impacts were not substantiated by reported changes in quantitative indicators.

## 4. Discussion

### 4.1. Does WASH FIT Improve WASH Outputs and Related Health Outcomes and Impacts? Evidence Is Inconclusive

The aim of this systematic review was to evaluate WASH FIT effectiveness, specifically by examining whether WASH FIT improves outputs for environmental health services and associated health outcomes and impacts. Overall, we found limited evidence suggesting that WASH FIT may improve environmental health service outputs and no robust evidence that WASH FIT improves health outcomes and impacts.

For studies of environmental health service outputs, only nine reported longitudinal data [28,30,31,34,36,37,40,44,46]. All of these studies found beneficial changes to outputs, such as improvements in the proportion of healthcare facilities with access to basic water, sanitation, and waste management infrastructure (e.g., [30,31,34,44]). Given that all nine of these studies consistently reported improvements from before to after implementation, it is plausible that WASH FIT improves environmental health service outputs (e.g., increased infrastructure access and functionality and improved behavioral practices). However, none of these studies compared progress against a control group for more robust evaluation. As such, improvements may alternatively be attributed to secular trends independent of WASH FIT programming.

A key impediment to our ability to evaluate WASH FIT effectiveness was missingness or ambiguity in reported outputs. We identified several shortcomings. One, studies reported data from the WASH FIT first edition assessment tool for “hygiene” or “sanitation” without specifying which specific subdomains were measured for hand hygiene, environmental cleaning, sanitation, or waste management [17,33,39]. This aggregation and ambiguity impeded our efforts to evaluate changes at the output level. The second edition of WASH FIT separates domains for hygiene, sanitation, cleaning, and waste management in the assessment tool, which should correct this issue. Two, studies indicated that they measured a domain but did not report data—or reported qualitatively that an output had changed but did not quantify specific metrics [28,29,34,45,46,47]. Three, studies did not indicate whether they measured a specific domain and reported neither quantitative nor qualitative data [15,28,30,31,40,42,43,47,48,49,50,51,52]. This may reflect a reporting deficiency or an adaptation to WASH FIT in which a particular domain was intentionally omitted from the assessment process. All of these reporting deficiencies undermined our efforts to evaluate WASH FIT effectiveness but could be addressed in future studies. To address incomplete or ambiguous reporting, reporting checklists can help ensure that all essential information is reported, aiding in the interpretation of individual studies and eventual meta-analyses [53,54].

For studies of health outcomes and impacts associated with WASH FIT, we found no meaningful evidence. In nearly all studies, health outcomes were reported qualitatively (n = 13, 42%) [30,32,36,37,38,43,44,45,48,49,50,51] or not at all (n = 12, 39%) [17,18,19,26,27,29,33,35,39,40,41,47,52]. Health impacts were hypothesized but never directly measured by any study. Our search yielded no experimental studies that robustly evaluated WASH FIT effectiveness on outputs, outcomes, or impacts. Without evidence from strong study designs that tracked longitudinal changes, we were unable to determine whether WASH FIT was effective in improving health outcomes and impacts.

We also identified inputs, activities, outputs, outcomes, and impacts using a logic model framework, and attempted to draw links between them to better understand the mechanisms by which WASH FIT may achieve change. We did not identify any studies that examined all components of the logic model or drew specific links between any components. Other studies have hypothesized logic models. Within the WASH FIT guide, the WHO and UNICEF propose five input categories (political; financial/material; human; civil society engagement; intersectoral collaboration: energy and climate, health), activities for the five-step WASH FIT cycle, five outcome categories (improved staff morale and performance; increased care seeking; improved infection prevention and control and reduced antimicrobial resistance; less environmental pollution and more sustainable waste management; more efficient use of resources and lower healthcare costs), and three long-term impacts (dignified, safe pregnancy and reduced maternal and newborn mortality; healthier, more productive families and communities; improved outbreak response and resilience) [11]. Weber et al. [20] proposed a conceptual evaluation framework for WASH FIT that closely follows the framework developed by WHO and UNICEF and includes additional outcomes for changes in finance and infrastructure operations and management. Our review identified similar categories, but overall, we found that the quality of evidence was too weak to corroborate either of these logic models.

Despite the lack of robust evidence, studies included in our review reflected positively on their experiences implementing WASH FIT and perceived changes in environmental conditions, which they attributed to WASH FIT. Continuous quality improvement principles (on which WASH FIT is based) have generally demonstrated favorable effects for other aspects of healthcare delivery, such as patient satisfaction and quality of care metrics [55,56,57]. Successes of other aspects of healthcare delivery suggest that the sustained use of WASH FIT for continuous quality improvement is a plausible mechanism to improve environmental health service outputs. However, we identified many studies that did not complete all five steps of the WASH FIT cycle, yet still attributed changes in outputs, outcomes, and impact to WASH FIT (e.g., [26,33,41]). This suggests that the underlying logic model may differ from what has been proposed by the WHO, UNICEF, and others.

Without sustained implementation beyond one-time assessments, continuous quality improvement principles are unlikely to explain any potential changes. In these cases, we hypothesize two potential explanations. First, WASH FIT may change to political will and incentivize investment. Multiple studies included in our review noted that WASH FIT assessments were used as an advocacy tool [32,36,37,38,50]. Stakeholders were convened and presented results of WASH FIT assessments that demonstrated poor environmental health services, demonstrating the need for improvements. In these cases, WASH FIT may galvanize funding and human resources needed to improve environmental health services, which are then delivered independently of WASH FIT. Second, existing studies on WASH FIT may suffer from chronological bias. In other words, it is plausible that the necessary components to improve environmental health services (e.g., funding, political will, human resources) trigger the adoption of WASH FIT programs, and improvements to environmental health services likely would have occurred regardless of whether WASH FIT was ever implemented in healthcare facilities. Because a majority of studies in our review were cross-sectional (n = 8, 26%) or case studies (n = 17, 55%) with no temporal component, it is not possible to attribute causality. These two explanations may also act together, where readiness for environmental health services interventions triggers the adoption of WASH FIT, which is subsequently used as an advocacy tool to secure further investment and further enhances readiness. In either case, WASH FIT’s promotion by the WHO and UNICEF lend legitimacy and likely have contributed to its widespread popularity, uptake, and perceived effectiveness [58,59].

Understanding the effectiveness of WASH FIT in healthcare facilities is critically important as it continues to grow in popularity and shape policies, priorities, and millions of dollars in investment. Given its influence, understanding if, how, and why it works should be prioritized for all stakeholders involved in funding, implementing, and promoting WASH FIT. Below, we make three specific recommendations for improving future studies and building evidence around WASH FIT effectiveness.

### 4.2. Recommendations for Improving Evidence Quality

#### 4.2.1. Disaggregate and Report Results of WASH FIT Assessments

By design, implementing WASH FIT will yield quantitative data for output indicators for environmental health services (i.e., the results of the WASH FIT assessment tool). The WASH FIT guide recommends that healthcare facilities iterate an assessment cycle every 6–12 months [11]. As these data should be collected and managed in such a way as to allow routine review as part of the recommended WASH FIT process, including them in published studies should be straightforward. Yet only half of included studies (n = 16) measured and reported quantitative data (i.e., WASH FIT indicator scores) for three or more WASH FIT domains or sub-domains [18,19,26,27,28,30,31,34,36,37,38,39,40,41,43,44].

We recommend that future studies disaggregate and report all WASH FIT assessment data. Documenting output changes is an essential preliminary step to understand whether and how downstream effects on health outcomes and impacts may occur. Furthermore, data collection and ongoing review are already essential components of the WASH FIT cycle, through assessments conducted under Step 2. As such, the effort required to track and report output-level changes should be minimal. Where programs intentionally modify or omit specific domains or indicators from their assessment and improvement plans, studies should explicitly describe the adaptation and rationale, such that results can be compared across studies.

#### 4.2.2. Identify Inputs, Activities, Outputs, Outcomes, and Impacts

We were unable to develop a robust logic model for WASH FIT, in part because of the ambiguous or incomplete reporting of inputs, activities, outputs, outcomes, and impacts across included studies. Understanding the logic model for WASH FIT is important for implementing and evaluating WASH FIT programs [60,61,62].

We recommend research to better define a logic model for WASH FIT. Prior research on costing may help identify inputs and activities. Bottom-up costing studies on environmental health services in healthcare facilities have developed frameworks to identify essential inputs and activities [63,64]. However, these frameworks are not tailored to WASH FIT programs and will require verification and likely adaptation. Process evaluation or similar techniques [65] to examine the implementation of WASH FIT programs will likely help develop and refine a logic model. Output indicators are the best established, with 95 indicators already available in the WASH FIT second edition assessment tool [11]. Outcome and impact indicators will prove more challenging to develop but are essential for rigorous effectiveness evaluation. The lack of reported outcomes and impacts in the studies included in our review likely reflects measurement challenges, such as the latency period between exposure to poor environmental health services and various health impacts—particularly infectious disease outcomes. Furthermore, the surveillance of healthcare-acquired infections and other relative rare outcomes is expensive and requires large sample sizes for evaluation [66]. Prior systematic reviews have similarly found insufficient evidence to assess the impacts of environmental health services on healthcare-acquired infections [2] and patient satisfaction and healthcare-seeking [4], in part because of these challenges. Qualitative and formative research has shown promise as a way to rapidly identify outcome and impact indicators that could be used in future WASH FIT evaluations [3,6].

#### 4.2.3. Conduct Experimental Studies and Exploit Natural Experiments

Experimental studies will be necessary to rigorously evaluate WASH FIT’s effectiveness in improving health outcomes and impacts. These studies will be costly. However, given the influence and investment in WASH FIT by countries—both countries adding line items for WASH FIT into their national health systems budgets and bilateral donors—we argue that they are important to ensure these investments in WASH FIT programs are effective and identify improvement opportunities.

In parallel to experimental studies, there are opportunities to exploit natural experiments among countries’ existing WASH FIT programs at considerably lower cost, by using national health management information systems. For example, reducing healthcare-acquired infections is routinely cited as a benefit of improving environmental health services by implementing WASH FIT. Five studies in our review claimed reduced healthcare-acquired infection as a benefit but did not substantiate this claim with evidence [17,19,39,41,44]. Many countries already monitor healthcare-acquired infections as part of their health management information systems. In countries implementing WASH FIT in a subset of regions or healthcare facilities within a region, matched controls could be identified among non-program areas. Quasi-experimental study designs could then be applied for a rigorous impact evaluation of WASH FIT using existing data. Other health outcomes and impacts could be assessed through the same approach (e.g., changes in patient volume or facility revenue to approximate changes in care seeking [67]), provided they are already incorporated into health information management systems.

We recommend that stakeholders incorporate more rigorous evaluation plans into WASH FIT programs. One possible solution to support countries and implementing partners is to incorporate evaluation guidelines into the WASH FIT implementation guide or create a companion guide as part of the suite of available WASH FIT materials. The WASH FIT manual focuses on applying data for improvement planning internal to the WASH FIT team and does not emphasize broader dissemination. Incorporating specific guidance would likely aid more generalizable evaluation and learning. WHO and UNICEF have created supplementary materials, including a training manual and technical fact sheets for WASH FIT [13]. An evaluation guide could supplement these materials.

### 4.3. Limitations

We limited our search to two peer-reviewed databases and one gray literature database. Washinhcf.org is the most comprehensive gray literature database for environmental health services in healthcare facilities, but it does not actively solicit studies. Our review captured available published studies, but there are likely many unpublished reports. Dedicated efforts to make those reports publicly available would strengthen the evidence base.

We recognize that definitions to classify outputs, outcomes, and impacts vary. Our review adapted definitions from the Kellogg Foundation [22]. We classified extracted data accordingly, but arguments could be made to classify some long-term outcomes (e.g., policy changes) as impacts. Vague descriptions hindered our efforts to extract data into the logic model framework in the literature. For example, studies that reported that a specific organization or government agency gave “support” were classified as inputs. However, the specific nature of this support was not clear.

## 5. Conclusions

This systematic review aimed to evaluate WASH FIT’s effectiveness at improving environmental health service outputs and associated health outcomes and impacts. A lack of quantitative data (particularly for outcomes and impacts), incomplete and inconsistent reporting, and weak study designs impeded this aim. Most studies reported outputs in terms of indicators measured as part of the WASH FIT assessment tool. Still, many of these assessments were performed to initiate the WASH FIT cycle and were not followed up with assessments after implementing WASH FIT improvement plans. We found no studies used a control group that would demonstrate change over time attributable to WASH FIT. WASH FIT may plausibly improve outputs. However, WASH FIT programs may reflect commitments and investments in environmental health services, yielding similar outputs even without WASH FIT. In either case, we cannot determine whether these outputs improve health outcomes and impacts.

Understanding whether and how WASH FIT achieves impact is important and will require future research. This evidence is important to ensure that funding invested for WASH FIT implementation is used cost-effectively and that opportunities to adapt and refine WASH FIT are fully realized as it continues to grow in popularity and influence. As a first step, we encourage more thorough reporting of all output indicators captured in WASH FIT assessments, particularly for follow-up and long-term monitoring after initial improvement plans are implemented. In the long term, we recommend experimental studies and exploiting natural experiments using data within health management information systems.

## Figures and Tables

**Table 1:** Categories of inputs for WASH FIT implementation.

Category	Definition	Examples
Financial Support	Financial or in-kind contributions of resources needed for WASH FIT implementation	Funds to establish the WASH FIT program (e.g., training of trainers, national-level orientation) [15,29,30]. Funds to make facility-level improvements (e.g., build infrastructure, purchase supplies) [19].
Human Resources	Recruitment and/or training of qualified personnel to implement WASH FIT	Recruiting technicians and engineers for infrastructure improvements [31]. Training WASH FIT team [17,26,39].
Technical Assistance	Subject-matter knowledge or expertise provided by an external entity to help address specific organizational needs	Supporting the Ministry of Health to co-develop a national infection prevention and control action plan [44]. Co-developing and leading a baseline assessment to target WASH FIT implementation with regional authorities [34].
Endorsements and commitments	Endorsement, encouragement, or approval of project activities by regulators or champions	Endorsement of activities and verbal encouragement to WASH FIT teams from national-level ministry staff [44]. The town council approved the indicators for the WASH FIT assessment [28].

**Table 2:** Categories of outputs and outcomes associated with WASH FIT implementation.

Category	Examples
Outputs	
Identification of WASH FIT gaps	Documenting the proportion of healthcare facilities meeting drinking water storage or bed spacing standards [33] Identifying healthcare facilities with lower levels of coverage for environmental health services compared to national and regional averages [19].
Personnel trained or mentored on WASH FIT	Training or mentoring of staff on WASH FIT implementation [38,43]. Recruiting and training technicians to implement environmental health service improvements [15].
Infrastructure coverage and access	Improving existing infrastructure operations and maintenance [28]. Constructing new or rehabilitating old infrastructure [31,36,40,44].
Outcomes	
Policy changes, including the decision to scale WASH FIT	Incorporating WASH FIT focal persons and improvement plans into national or regional standards for a minimal package of essential health services [30]. Scaling up WASH FIT into the national strategy for the Ministry of Health [44]. Incorporating indicators for WASH FIT implementation into national health information monitoring systems [42].
Coordination, collaboration, and feedback	Enhancing collaboration between local government officials and healthcare facility-level administrators [25,29].
Funding for WASH FIT and environmental health services	Advocating at the national and regional levels for greater funding for WASH FIT and environmental health services [25,44]. Incorporating WASH FIT into budgets at the healthcare facility level [44].
Knowledge, attitudes, and practices among healthcare staff	Improving staff motivation and satisfaction [30,44]. Improving staff practices for infection prevention and control and waste management [44].
Knowledge, attitudes, and practices among patients/community members	Improving staff practices for waste disposal in designated bins [34]. Increasing community members’ risk awareness and willingness to report issues to the WASH FIT team [28].
Community engagement	Helping create infection prevention and control committees that included diverse participants and community members [36]. Engaging community members on the WASH FIT team [34].

## Data Availability

The original contributions presented in this study are included in the article/supplementary material. Further inquiries can be directed to the corresponding author.

## Supplementary Materials

The following supporting information can be downloaded at https://www.mdpi.com/article/10.3390/ijerph22050708/s1, Table S1: PRISMA checklist; Table S2: Extraction forms.
